# Posterior Neck Pain as the Initial Manifestation of Giant Cell Arteritis: A Diagnostic Challenge

**DOI:** 10.7759/cureus.116951

**Published:** 2026-09-25

**Authors:** Thejas Swaroop Konduru, Sharada Medapuram Belagu, Nithyananda Rajaiah

**Affiliations:** 1 Acute Internal Medicine, Midland Metropolitan University Hospital, Sandwell and West Birmingham NHS Trust, Birmingham, GBR; 2 Respiratory Medicine, Walsall Manor Hospital, Walsall, GBR; 3 Acute and Internal Medicine, Sandwell and West Birmingham NHS trust, Birmingham, GBR

**Keywords:** doppler us, giant cell arteritis (gca), neck pain, occipital headache, temporal artery biopsy

## Abstract

Giant cell arteritis (GCA) is a granulomatous large-vessel vasculitis that typically affects the temporal arteries, presenting with temporal headache, jaw claudication, or visual symptoms. However, not all cases follow the classical pattern. We report a rare case of a 75-year-old woman who presented solely with isolated occipital headache and posterior neck pain, with no temporal tenderness, jaw claudication, or visual complaints. On examination, both temporal arteries were thickened and pulseless. Vascular imaging revealed non-compressible circumferential wall thickening and severe luminal occlusion involving the bilateral superficial temporal main trunks as well as their frontal and parietal branches. This striking dissociation between clinical symptoms and vascular involvement highlights that GCA can silently involve the temporal arteries while presenting atypically, underscoring the importance of considering GCA even when the only symptom is occipital headache.

## Introduction

Giant cell arteritis (GCA) is a granulomatous vasculitis primarily affecting large and medium-sized arteries, especially the cranial branches of the external carotid artery [1]. It typically presents in individuals over 50 years of age and has a female predominance [2]. Classical symptoms include new-onset temporal headache, scalp tenderness, jaw claudication, and visual disturbances, often accompanied by elevated inflammatory markers and polymyalgia rheumatica (PMR) [1,2].

The 1990 American College of Rheumatology (ACR) criteria for classification of GCA include age ≥50 years, new headache, temporal artery tenderness or reduced pulsation, erythrocyte sedimentation rate (ESR) ≥50 mm/h, and abnormal temporal artery biopsy findings [3]. While specific, these criteria may not fully encompass atypical or extracranial presentations [3].

Occipital artery involvement is an uncommon manifestation and may present solely as occipital headache or posterior neck pain [4-6]. In such cases, physical examination of temporal arteries may be unremarkable. Increasingly, imaging modalities such as Doppler ultrasound are being used to detect vascular changes even in the absence of clinical signs [7].

We present a rare case of GCA in a 75-year-old woman who exhibited isolated occipital headache and posterior neck pain without classical cranial features. Despite the lack of temporal pain or visual complaints, Doppler ultrasound strongly supported the diagnosis by demonstrating near-complete occlusion of all three major branches of both temporal arteries. This highlights the diagnostic value of early vascular imaging in atypical presentations.

## Case presentation

A 75-year-old female with a past medical history of hypercholesterolemia presented with a two-week history of posterior neck pain and a sudden-onset occipital headache that had progressively worsened over 10 days. She denied symptoms suggestive of raised intracranial pressure, jaw claudication, visual disturbances, dental pain, or recent trauma. She reported two episodes of non-projectile vomiting, attributed to analgesic use (codeine with paracetamol), which she subsequently discontinued. Her surgical history included a hysterectomy for uterine prolapse. She was taking atorvastatin and was otherwise not on regular medications. She had no history of smoking and no significant family medical history.

On examination, her vital signs were stable, and she was afebrile. Neurological assessment, including visual examination, was unremarkable. She had marked tenderness over the cervical spine; however, there was no radicular arm pain, no sensory or motor deficit in a C7 distribution, and negative nerve root tension signs were observed. Although there was no scalp tenderness, both temporal arteries were noted to be thickened and pulseless bilaterally.

Initial laboratory investigations revealed normal-to-low leukocyte counts alongside disproportionately high erythrocyte sedimentation rate (ESR) and C-reactive protein (CRP) levels (Table 1). Routine biochemistry confirmed normal baseline organ function and the absence of monoclonal gammopathy. Minor incidental elevations in thyroid-stimulating hormone (TSH) and parathyroid hormone (PTH) were noted but were consistent with subclinical hypothyroidism and age-related vitamin D deficiency, given normal free T4 and calcium levels. Electrocardiography showed sinus rhythm with left axis deviation. A non-contrast computed tomography (CT) scan of the head revealed no acute intracranial pathology.

**Table 1:** Laboratory investigations at initial presentation. INR: international normalized ratio; eGFR: estimated glomerular filtration rate

Laboratory tests	Parameters	Value	Reference range
Full blood count	White blood cells (x10^9^/L)	3.6	4-11
	Red blood cells (x10^12^/L)	4.63	4.5-6
	Hemoglobin (g/L)	140	117-161
	Hematocrit (L/L)	0.43	0.4-0.5
	Neutrophils (x10^9^/L)	1.86	1.70-7.50
	Lymphocytes (x10^9^/L)	1.28	1-4
	Monocytes (x10^9^/L)	0.39	0.2-0.8
	Basophils (x10^9^/L)	0.09	0-0.10
	Eosinophils (x10^9^/L)	0.02	0.1-0.4
	Platelets (x10^9^/L)	183	150-400
	ESR (mm/h)	117	<30
Hematinics	Ferritin (µg/L)	160	22-275
	Vitamin B12 (ng/L)	>128	51-128
	Folate (µg/L)	6.7	3.1-20
Coagulation screen	INR	1.1	0.80-1.20
Urea and electrolytes	Sodium (mmol/L)	142	135-145
	Potassium (mmol/L)	4.9	3.5-5.5
	Urea (mmol/L)	6	2.5-7.8
	eGFR (mL/min/1.73 m^2^)	82	>90
	Creatinine (µmol/L)	62	59-104
	Calcium (albumin adjusted) (mmol/L)	2.36	2.2-2.6
Inflammatory markers	CRP (mg/L)	202	<5
Lipids	Total cholesterol (mmol/L)	4.9	2.5-5
	High-density lipoprotein (mmol/L)	1	>1.2
	Triglycerides (mmol/L)	1.5	<2.3
Endocrine	Thyroid-stimulating hormone (mIU/L)	8.31	0.35-4.94
	Free thyroxine (T4) (pmol/L)	10	9-20
	Parathyroid hormone (pmol/L)	21.4	2-11.3
Liver function tests	Bilirubin (µmol/L)	7	<21
	Aspartate aminotransferase (U/L)	20	<37
	Alanine aminotransferase (U/L)	14	<45
	Alkaline phosphatase (U/L)	102	20-130
	Gamma-glutamyl transpeptidase (U/L)	19	<45
	Albumin (g/L)	40	35-50
Autoimmune screen	Antinuclear antibody	Negative	
	Extractable nuclear antigen	Negative	
	Anti-neutrophil cytoplasmic antibody	Negative	

Given the elevated inflammatory markers and the absence of a clear etiology, rheumatology consultation was sought for consideration of an atypical presentation of GCA. A decision was made to administer a single dose of intravenous methylprednisolone (500 mg). Following corticosteroid administration, the patient experienced some improvement in headache symptoms, although her neck pain persisted. Oral prednisolone was initiated at a dose of 1 mg/kg. Ophthalmology review did not reveal any ocular involvement.

Due to ongoing cervical pain, differentials such as cervical discitis or a localized spinal collection were considered. Magnetic resonance imaging (MRI) of the cervical spine showed disc herniation and foraminal stenosis at C6-C7 levels without evidence of nerve root compromise. Because the patient had no focal radicular symptoms and her pain ultimately resolved completely following continued corticosteroid therapy, the MRI findings were considered incidental degenerative changes rather than the primary cause of her symptoms.

A Doppler ultrasound of the temporal arteries demonstrated non-compressible circumferential wall thickening (halo sign), elevated intima-media thickness (IMT), and a lack of luminal Doppler flow, indicating severe luminal occlusion involving the bilateral superficial temporal main trunks as well as their frontal and parietal branches (Figure 1). Bilateral axillary arteries were also scanned and appeared normal without vasculitic changes. These findings strongly supported the clinical suspicion of GCA. A temporal artery biopsy was deferred because high-resolution Doppler ultrasound clearly demonstrated classical signs of GCA in the setting of severe inflammatory marker elevation and marked clinical response to steroids, in accordance with current European Alliance of Associations for Rheumatology (EULAR) recommendations prioritizing non-invasive ultrasound as the primary diagnostic tool.

**Figure 1:**
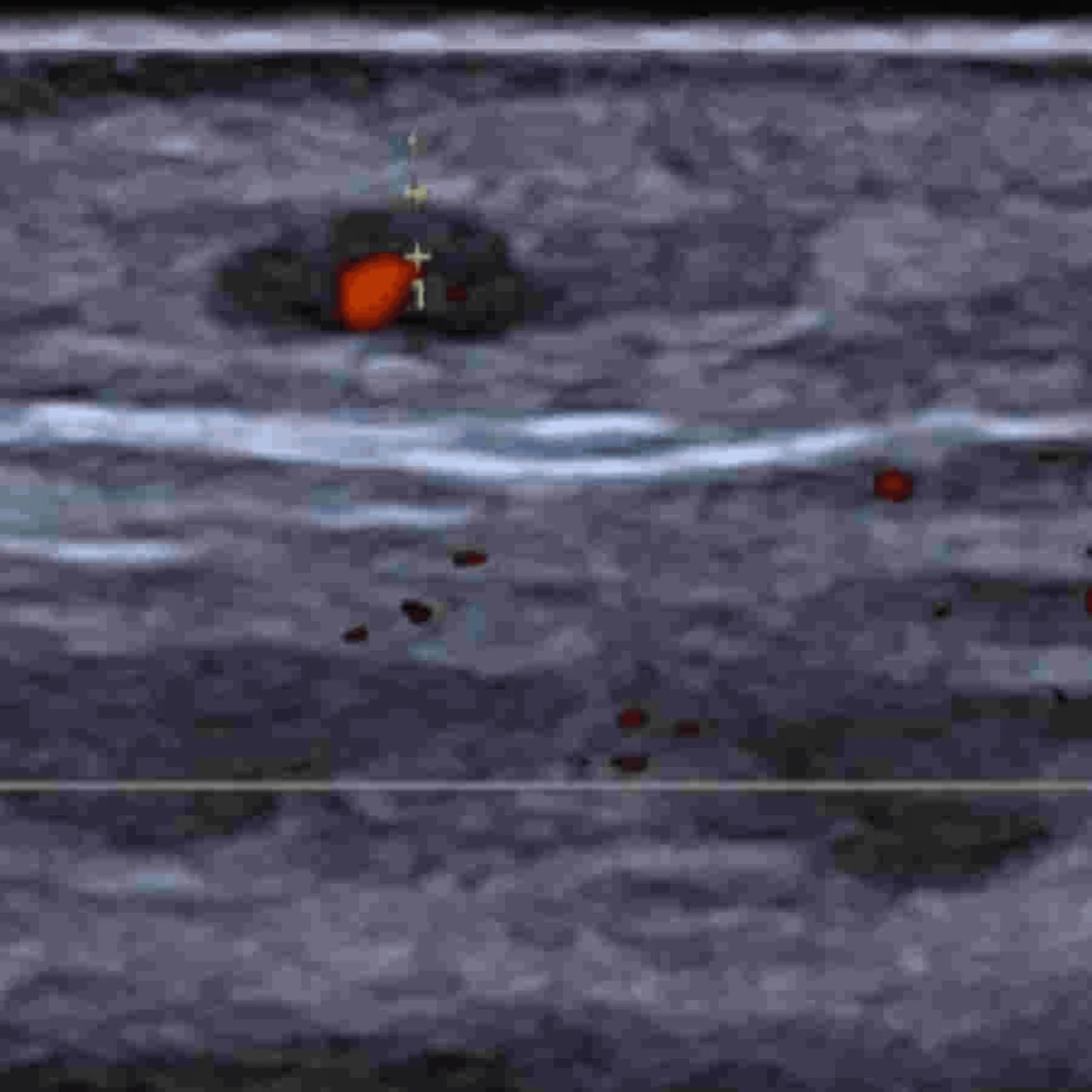
Transverse color Doppler ultrasonography of the temporal artery. The ultrasound demonstrates a characteristic halo sign, non-compressible vessel wall thickening, and lack of luminal flow, indicating near-complete occlusion of the main superficial temporal artery trunk and its frontal and parietal branches.

The patient was commenced on low-dose aspirin (75 mg daily) alongside continued corticosteroid therapy. Her dual-energy X-ray absorptiometry (DEXA) scan showed osteopenia, and she was commenced on bone protection therapy. At a four-week follow-up in the rheumatology clinic, she reported marked clinical improvement. The headache and neck pain had fully resolved, confirming the inflammatory (rather than mechanical) etiology of her symptoms. On examination, temporal arteries were soft and non-tender, with feebly palpable pulses. Repeat inflammatory markers demonstrated a significant downward trend, and she was advised to continue follow-up with rheumatology.

## Discussion

Giant cell arteritis is the most prevalent systemic vasculitis in adults over 50 years of age, most often involving the superficial temporal artery and its branches. Early diagnosis is critical to prevent severe complications such as irreversible vision loss and stroke [1,2]. While the classic clinical picture includes temporal headache, jaw claudication, and visual disturbances, atypical forms of GCA are increasingly recognized. Isolated occipital pain, as seen in our patient, is a less common but documented presentation [5,6].

The striking dissociation between occipital pain and temporal artery disease in this case warrants discussion. Occipital headache in GCA can stem from direct involvement of the occipital artery (a branch of the external carotid artery) or referred pain via shared cervical dermatomes and cranial nerve vascular innervation. While the temporal branches were occluded without local pain, the occipital pain likely represented referred vascular pain or concomitant subclinical occipital arteritis, compounded by the absence of classical temporal region tenderness.

Doppler ultrasound has become a valuable non-invasive tool in the evaluation of suspected GCA. Characteristic features such as the "halo sign," wall thickening, and arterial occlusion are considered highly specific [7,8]. In our patient, Doppler imaging provided a definitive clue even when classical cranial features were missing.

Traditional reliance on the 1990 American College of Rheumatology (ACR) criteria may limit the detection of such atypical cases. The more recent 2022 ACR/EULAR criteria emphasize the role of imaging; however, it is vital to note that these are classification criteria for research purposes, whereas clinical diagnosis relies on the synthesis of history, physical exam, inflammatory markers, imaging, and treatment response. Applying the 2022 ACR/EULAR classification criteria to our patient yields a score of 12 points as follows: age ≥50 years, morning stiffness/neck pain, maximum ESR ≥50 mm/h, temporal artery thickening/pulselessness, and positive vascular imaging with halo sign/occlusion.

This easily surpasses the threshold of ≥6 points for classification as GCA, further corroborating the clinical diagnosis. This case underscores the importance of maintaining diagnostic vigilance for GCA in elderly patients presenting with new-onset headaches in atypical locations. Incorporating vascular imaging early can guide timely corticosteroid initiation and reduce the likelihood of ischemic complications.

## Conclusions

This case describes a 75-year-old woman who presented with isolated occipital headache and posterior neck pain without temporal headache, jaw claudication, visual symptoms, or scalp tenderness, despite having extensive bilateral temporal artery occlusion demonstrated on Doppler ultrasound. The marked discordance between the clinical presentation and the severity of temporal artery involvement illustrates that giant cell arteritis may present with atypical symptoms while significant cranial vascular disease remains clinically silent. Recognition of the abnormal temporal artery examination and timely vascular imaging enabled prompt diagnosis and initiation of corticosteroid therapy, resulting in full clinical improvement and confirming the inflammatory nature of her cervical pain. This case highlights the importance of maintaining a high index of suspicion for giant cell arteritis in older patients with new-onset atypical headache when inflammatory markers are elevated and examination findings raise concern, even in the absence of classical cranial features.

## References

[1] Pepper K (2023). Giant cell arteritis. Postgrad Med.

[2] Weyand CM, Goronzy JJ (2014). Giant-cell arteritis and polymyalgia rheumatica. N Engl J Med.

[3] Hunder GG, Bloch DA, Michel BA (1990). The American College of Rheumatology 1990 criteria for the classification of giant cell arteritis. Arthritis Rheum.

[4] Prieto-Peña D, Castañeda S, Martínez-Rodríguez I, Atienza-Mateo B, Blanco R, González-Gay MA (2021). Imaging tests in the early diagnosis of giant cell arteritis. J Clin Med.

[5] Pinnell J, Tiivas C, Perkins P, Blake T, Saravana S, Dubey S (2018). Ultrasonography of occipital arteries to diagnose giant cell arteritis: a case series and literature review. Clin Rheumatol.

[6] Guggenberger KV, Riedling L, Kern D (2025). Isolated inflammatory involvement of the occipital artery in giant cell arteritis and polymyalgia rheumatica: findings from a retrospective analysis and the critical role of MRI in diagnosis. Rheumatol Int.

[7] Schmidt WA, Kraft HE, Vorpahl K, Völker L, Gromnica-Ihle EJ (1997). Color duplex ultrasonography in the diagnosis of temporal arteritis. N Engl J Med.

[8] Hellmich B, Agueda A, Monti S (2020). 2018 update of the EULAR recommendations for the management of large vessel vasculitis. Ann Rheum Dis.

